# Filaria Sanguinis Hominis

**Published:** 1882-05

**Authors:** 


### FILARIA SANGUINIS HOMINIS.

From the London *Lancet* we note that, in the twenty-first issue of the Chinese Customs Medical Reports, Dr. Myers records some further interesting observations concerning the filaria sanguinis hominis, made in the island of Formosa. It seems, that although this island is only separated from the main-land, where filaria disease abounds, by a channel 180 miles wide, three cases only of elephantoid disease have presented themselves in nine years among 15,000 general hospital patients, and the three filaria-infested persons Dr. Myers saw all came from the main-land (Amoy and district.) This fact, that the disease does not spread in the island, as Manson conjectured it might have done into Barbadoes, is the most remarkable, as communication with the main-land is constant, and there is an abundance of mosquitoes ; so that Dr. Myers surmises that the particular species of mosquito which is the true intermediary host of the filaria exists on the main-land, and probably not on the island. He is at present carrying out a systematic investigation of the different species of mosquito. First, Dr. Myers put a conveniently willing and infected man to sleep in a gauze-covered mosquito-house, into which he turned mosquitoes collected promiscuously from all parts of Formosa. These gorged insects he examined day by day, and found that the next morning after their feed they contained several lively embryos, which at later date became digested, and had evidently not got into their true host, for Manson has found that mosquitoes containing filaria sanguinis hominis, when fed on an infected dog, digested the filaria of the latter. Secondly, Dr. Myers has also made some observations on the periodicity of the appearance and disappearance of the filaria embryos in respect of the blood-circulation, in the case of a man suffering from glandular swellings and recurrent lymphatic fever, but otherwise healthy. The tables given show a result in complete accord with Manson's observations. The embryos appeared in the blood-circulation regularly between 6 P.M. and 8 P.M., generally a little after 6 P.M., and were present in greatest numbers about midnight, after which they gradually decreased, and pretty well disappeared by 6 A.M. to 8 A.M. This period of twelve hours is just the time that, as Manson has shown, the mosquito is in active search for food. Thirdly, Dr. Myers tries to make out what becomes of the embryos when they disappear from the blood-circulation—whether this disappearance is final as regards the swarm, and caused by death, or whether they only lie dormant for the time, perhaps in some viscera, as the lungs. He made prolonged and careful observations on the condition of the embryos at the times of their appearance in the circulation and their disappearance, and he found that the most marked and unmistakable contrast existed between their excessively active, vigorous condition at the former time and their increasingly torpid and feeble, shrivelled, straightened out state at the latter time. He thinks the disappearance is final as regards the swarm, and he further argues that unless the parent or parents breed only once, or at long intervals, the circulation would get almost blocked by the myriads of filariæ if there were not some more wholesale method of removal than mosquito-sucking. Dr. Myers suggests the possibility of the diurnal solution of dead embryos. Fourthly, Dr. Myers watched the behavior of the embryos with such substances as bisulphate of quinine, salicylic acid, arsenious acid, and santonin. The quinine affected them most, but the upshot of the matter is that the quantity of any of these substances necessary to kill the embryos would kill the host also. The problem, therefore, seems to be how to diagnose the exact situation of the parent-worm (generally lying in the superficial glands) with an accuracy sufficient to extract it.—*Medical and Surgical Reporter.*

